# Interaction of toxic and non-toxic HypF-N oligomers with lipid bilayers investigated at high resolution with atomic force microscopy

**DOI:** 10.18632/oncotarget.10449

**Published:** 2016-07-06

**Authors:** Reinier Oropesa-Nuñez, Silvia Seghezza, Silvia Dante, Alberto Diaspro, Roberta Cascella, Cristina Cecchi, Massimo Stefani, Fabrizio Chiti, Claudio Canale

**Affiliations:** ^1^ Department of Nanophysics, Istituto Italiano di Tecnologia, Genova, Italy; ^2^ DIBRIS Department, University of Genova, Genova, Italy; ^3^ Department of Physics, University of Genova, Genova, Italy; ^4^ Section of Biochemistry, Department of Biomedical Experimental and Clinical Sciences, University of Florence, Firenze, Italy

**Keywords:** protein misfolding, protein aggregation, ganglioside GM1, amyloid oligomers, AFM

## Abstract

Protein misfolded oligomers are considered the most toxic species amongst those formed in the process of amyloid formation and the molecular basis of their toxicity, although not completely understood, is thought to originate from the interaction with the cellular membrane. Here, we sought to highlight the molecular determinants of oligomer-membrane interaction by atomic force microscopy. We monitored the interaction between multiphase supported lipid bilayers and two types of HypF-N oligomers displaying different structural features and cytotoxicities. By our approach we imaged with unprecedented resolution the ordered and disordered lipid phases of the bilayer and different oligomer structures interacting with either phase. We identified the oligomers and lipids responsible for toxicity and, more generally, we established the importance of the membrane lipid component in mediating oligomer toxicity. Our findings support the importance of GM1 ganglioside in mediating the oligomer-bilayer interaction and support a mechanism of oligomer cytotoxicity involving bilayer destabilization by globular oligomers within GM1-rich ordered raft regions rather than by annular oligomers in the surrounding disordered membrane domains.

## INTRODUCTION

The aberrant self-assembly of peptide/protein molecules in the intra/extracellular space of tissues and organs to form highly structured fibrillar aggregates is associated with a group of highly debilitating pathological conditions including Alzheimer's and Parkinson's disease, familial amyloid polyneuropathy, spongiform encephalopathies, type II diabetes, systemic amyloidoses and many others [1–4]. In such diseases, particularly in neurodegenerative conditions, it is thought that an important cytotoxic role is played by small protein oligomers arising during peptide/protein aggregation. These toxic species form transiently during the process of aggregation as on- or off-pathways entities [5, 6], but can also be released from, or generated by, the fibrillar deposits that accumulate as end products in organs and tissues [7–9].

Studies on the N-terminal domain of the *E. coli* HypF protein (HypF-N) have contributed to our knowledge of the structural properties of these putatively pathogenic oligomers and of the mechanisms by which they cause toxicity to exposed cells [10–19]. Indeed, HypF-N is particularly useful to characterize the properties and the structure-toxicity relationship of misfolded oligomers for a number of reasons. First, HypF-N forms spherical oligomers and amyloid-like fibrils *in vitro*, under conditions that destabilize its native structure [14, 20–23]. Second, HypF-N oligomers formed *in vitro* and added to the extracellular medium of cultured cells, [10–12, 14–17, 19] or injected into rat brains [13, 15] impair cell viability similarly to oligomers found in many amyloid diseases. Third, HypF-N oligomers bind to cultured primary rat neurons and co-localise with post-synaptic densities, inhibit long-term potentiation (LTP) in rat hippocampal slices and induce cognitive impairment following their injection into rat brains, thus producing all the effects of the Aβ_42_ oligomers associated with Alzheimer's disease at the biochemical, biological and electrophysiological levels [18]. Fourth, HypF-N can be purified to a high yield with relatively low costs and provides highly reproducible results in studies on protein aggregation and on the toxicity of the resulting oligomers [14, 15, 17, 24]. Fifth, HypFN is a bacterial protein not associated with amyloid diseases and for this reason provides general data that do not depend on possible modifications of specific cellular proteins undergoing aggregation. Finally, and very importantly, HypF-N oligomers are stable and maintain their morphological and structural properties even under conditions very different from those that promoted their formation, allowing their detailed study at the structural and biological level [14–16, 18, 19].

Two protocols have been established to form two different types of stable HypF-N oligomers, named type A (OA) and type B (OB), respectively [14]. These oligomeric forms share a similar size and morphology when observed by atomic force microscopy (AFM). In fact, both types are roughly spherical, with a diameter of 2-6 nm and bind thioflavin T (ThT) with similar enhancement of fluorescence, yet reduced with respect to that commonly observed for mature amyloid fibrils [14]. In spite of their common morphological and tinctorial properties, only OA display significant toxicity to cultured neuronal cells, cultured primary neurons and whole animal models [14–16, 18]. In neuroblastoma cells, such a toxicity resulted from the interaction of the oligomers with the cell membrane with consequent Ca^2+^ influx from the extracellular space to the cytosol [15]. This early event triggers a complex cascade of effects, including an increase of intracellular ROS levels, cellular internalization of oligomers across the membrane, caspase-3 activation, mitochondrial sufferance, etc. resulting in cell death by apoptosis or necrosis [25] None of such effects was observed for OB, in spite of the observation that both OA and OB were able to physically interact with the cell membrane. This suggested that the very different biological effects of OA and OB could be the result of the different features of their initial interaction with the cell membrane [15].

ANS binding and site-directed pyrene labelling experiments led to propose that the different toxicity of OA and OB was attributable to the higher solvent-exposure and flexibility of hydrophobic amino acid residues located on the surface of the former with respect to the latter [14]. Later on, it was found that OA toxicity also depended on the lipid composition of the phospholipid bilayer. In particular, OA toxicity appeared to be modulated by the content of the ganglioside GM1, as its depletion from, or further addition to, neuroblastoma cells resulted in a dramatic loss or exacerbation of OA toxicity, respectively. Interestingly, cell enrichment with GM1 caused the normally non-toxic OB to become toxic, whereas cell depletion of GM1 caused the normally toxic OA to become non-toxic [16]. Overall, these results indicated that oligomer toxicity depends not only on specific structural properties of the oligomers themselves but also on the biochemical and biophysical properties of the membrane they interact with, in a delicate and complex interplay between the structural and physicochemical features of both [26]. Furthermore, these considerations add to, and confirm, previous and recent data indicating that GM1 is a pivotal player in Aβ peptide neurotoxicity both by contributing to Aβ oligomer nucleation and growth into toxic fibrils [27, 28] and by sequestering Aβ oligomers from brain interstitial fluid onto neuronal membranes [29].

Since the interaction between HypF-N oligomers and the cell membrane is a central event of the resulting toxicity cascade, in the present study we sought to provide further information on this interaction. To this aim, we used both OA and OB and supported lipid bilayers (SLBs) that lacked, or contained a physiological content of GM1, and monitored the resulting interactions by AFM. By this approach we were able to isolate the contribution of the lipid component of the plasma membrane, separating it from that of the protein component in the evaluation of the oligomer-membrane interaction. It also allowed the imaging of such an interaction at the sub-microscopic level with high and unprecedented resolution and the discrimination between ordered and disordered lipid phases in the bilayer, which mimics the important distinction between lipid rafts and the surrounding disordered plasma membrane domains in natural cells.

## RESULTS

### Interaction between toxic and non-toxic HypF-N oligomers and lipid bilayers

HypF-N oligomers were preformed under two different conditions, traditionally referred to as condition A and condition B, as previously reported [14]. We will refer to the two types of oligomers as OA and OB, respectively. We formed separately model membranes as supported lipid bilayers (SLBs) composed of 1,2-dioleoyl-sn-glycero-3-phosphocholine (DOPC), sphingomyelin (SM), cholesterol (chol) with or without the ganglioside GM1 (DOPC:SM:chol:GM1 and DOPC:SM:chol, respectively). Therefore, two different lipid mixtures, GM1-enriched or GM1-free, were used (+GM1 or −GM1, respectively).

**Figure 1:**
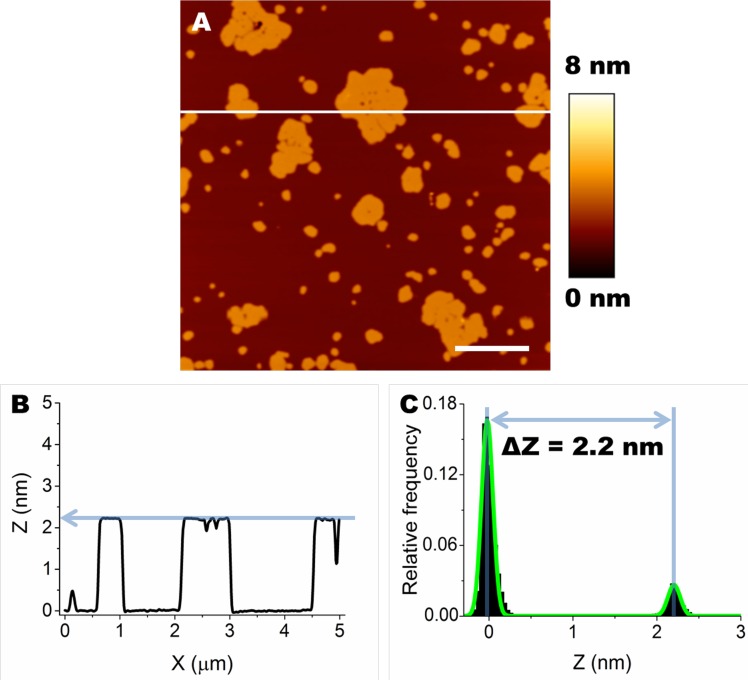
Formation of L_**α**_ and L_**β**_ domains by segregation of lipid species in SLBs **A.** Typical morphology of SLBs containing GM1 (DOPC:SM:chol:GM1). Scale bar: 1.0 μm. **B.** A cross section was taken along the white line in A (X axis). The difference in height between L_β_ and L_α_ is ≈ 2.2 nm. **C.** The height distribution of Z values, fitted with the sum of two Gaussian curves (green line), shows the difference in thickness (∆Z) between L_β_ and L_α_ domains.

SLBs prepared on mica from either lipid mixture displayed segregation of different lipid species in well distinct, differently ordered, domains. In particular, domains with low level of order, generally considered as fluid domains (L_α_ phase domains), were composed of DOPC. SM, chol and GM1 (when present) segregated in areas with a higher level of order, considered as gel phase domains (L_β_ phase domains) [30]. As expected, L_β_ and L_α_ domains also displayed different thickness; in particular, we consider ΔZ = Z_Lβ_ − Z_Lα_, where Z_Lβ_ and Z_Lα_ are the thickness of the ordered and disordered phases, respectively [30–32]. Since the Z_Lα_ was taken as our reference, it was given arbitrarily the value of 0 nm (Z_Lα_ = 0 nm). Consequently, ΔZ = Z_Lβ_. Our analysis can be appreciated in the example shown in Figure 1A. A single line profile, corresponding to the white line indicated in Figure 1A, is shown in Figure 1B. An average value of ΔZ was calculated for each image considering the separation between the two peaks of the distribution of Z values fitted with the sum of two Gaussian curves (Figure 1C).

**Figure 2:**
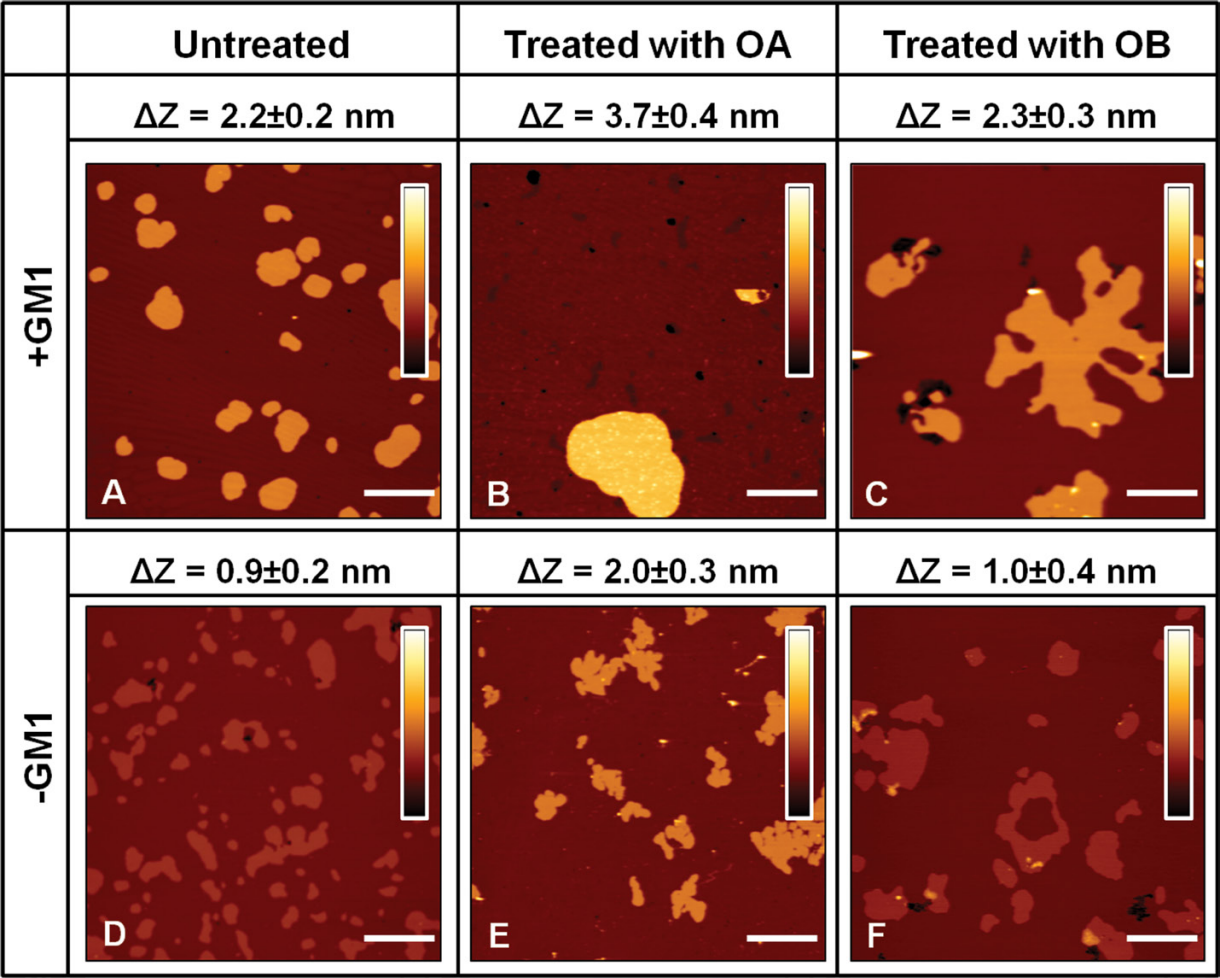
SLBs imaging in the presence A.-C. or in the absence D.-F. of GM1 The images were acquired on bilayers that were left untreated **A.**, **D.**, treated with OA **B.**, **E.** or treated with OB **C.**, **F.**, respectively. The height difference (∆Z) between L_β_ and L_α_ phases was estimated for each case by averaging the distance between Gaussian peaks in pixel height distributions associated with a variable number of images (n≥10). For both lipid mixtures, ΔZ increases after the administration of 12 μM OA **B.**, **E.** and is preserved after the administration of the same concentration of OB **C.**, **F.**. The colour bars correspond to a *Z* range of 8.0 nm. Scale bars: 1.0 μm.

ΔZ values for all the analysed conditions were obtained from the analysis of a variable number of images (n≥10). ΔZ was extremely reproducible, resulting in a value of 2.2±0.2 nm in the presence of GM1 (Figure 2A). The administration of 12 μM OA to SLBs containing GM1 affected lipid organization, inducing a significant increase in ΔZ to a value of 3.7±0.4 nm (Figure 2B). The same behaviour was also observed on bilayers devoid of GM1. However, in this case the value of ΔZ was found to be 0.9±0.2 nm in the absence of protein oligomers (Figure 2D) and rose to 2.0±0.3 nm after oligomer administration (Figure 2E). A different scenario was found when lipid membranes were treated with non-toxic OB. In fact, in this case the ∆Z values measured after oligomer administration, 2.3±0.3 nm in the presence of GM1 (Figure 2C) and 1.0±0.4 nm in the absence of GM1 (Figure 2F), were not significantly different from those measured in the absence of oligomers (Figure 2A, 2B). In conclusion, only the addition of the toxic OA oligomers resulted in a remarkable increase of the difference in thickness between lipid phases of both GM1-enriched and GM1-free lipid membranes.

**Figure 3:**
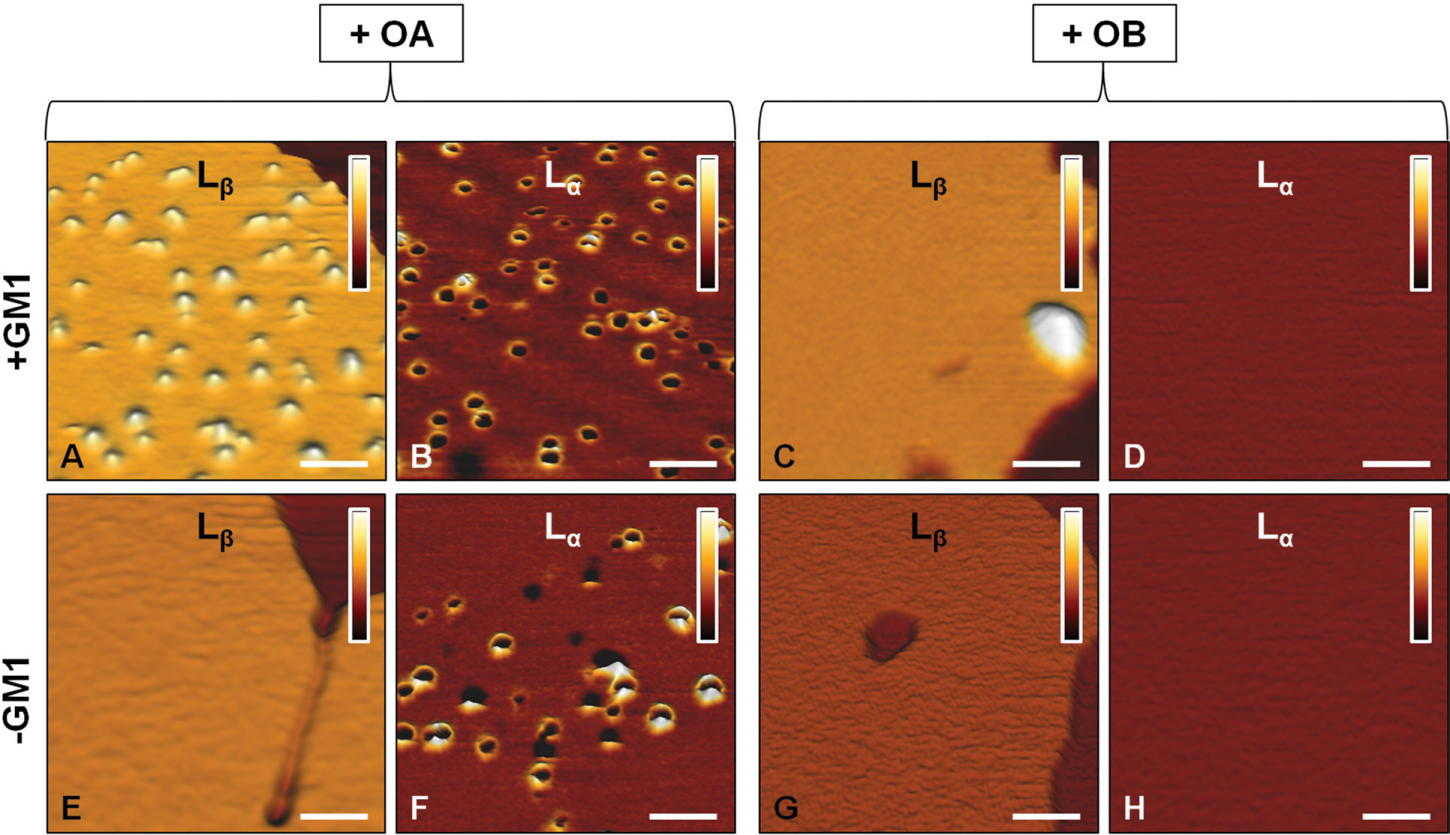
AFM images at higher lateral resolution L_β_ and L_α_ phases of SLBs formed in the presence **A.**-**D.** or in the absence **E.**-**H.** of GM1 and after the administration of 12 μM OA **A.**,**B.**,**E.**,**F.** or OB **C.**,**D.**,**G.**,**H.**. Globular aggregates appear on the L_β_ phase only in the presence of GM1 and after administration of OA **A.** Annular aggregates appear on the L_α_ phase of both GM1-enriched and GM1-depleted SLBs after administration of OA **B.** and **F.**. OB interact scarcely with both L_α_ and L_β_ phases of both GM1-enriched and GM1-free SLBs **C.**,**D.**,**G.**,**H.**. Only a small number of large aggregates is observed on the L_β_ phase in the presence of GM1 **C.** The colour bars correspond to a *Z* range of 5.0 nm. Scale bars: 100 nm.

**Figure 4:**
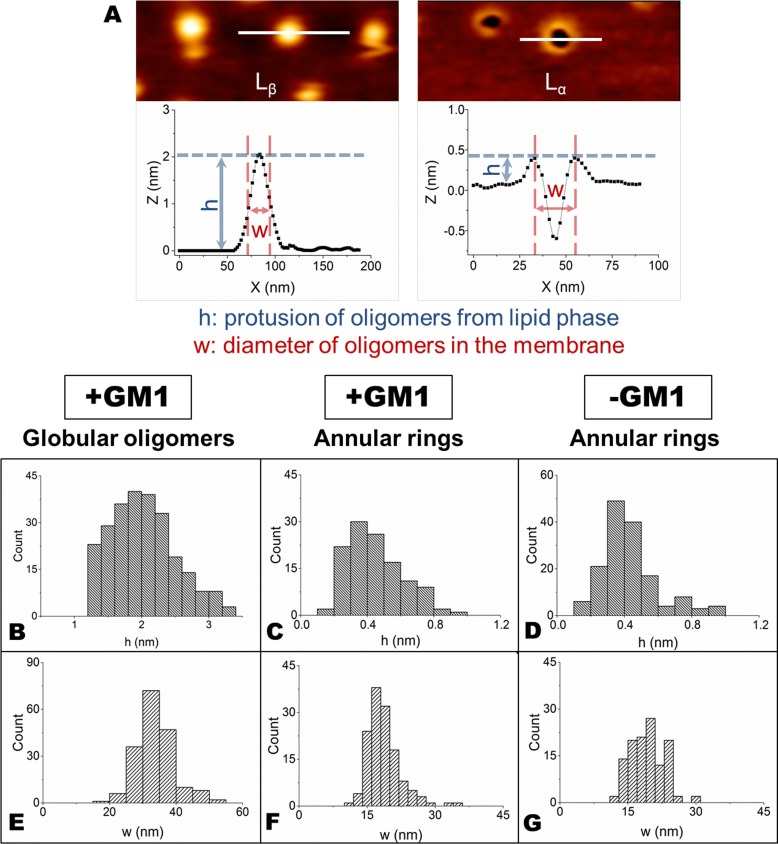
**A.** The width (w) of globular oligomers in the L_β_ phase was measured at half height of the aggregates, while the width (w) of annular rings in the L_α_ phase represents the diameter of the circular structure, calculated as shown in the figure. The height (h) of both aggregates was determined as the difference of height between the L_α_ or L_β_ phase and the top of the aggregates. **B.**-**G.** Distributions of h **B.**,**C.**,**D.** and w **E.**,**F.**,**G.** values for globular **B.**,**E.** and annular **C.**,**D.**,**F.**,**G.** structures calculated on GM1-enriched **B.**,**C.**,**E.**,**F.** and GM1-depleted **D.**,**G.** SLBs.

An important insight in the mechanism of interaction of OA with SLBs was deduced from images acquired at higher lateral resolution. In the presence of GM1, OA interact, though differently, with both lipid phases (Figure 3A, 3B). In particular, an accumulation of globular aggregates, protruding by 2.0±0.5 nm from the membrane surface, was observed on the L_β_ phase of GM1-enriched lipid membranes (Figure 3A). Since the size of OA is 2-6 nm, as estimated in previous studies [14, 17], we conclude that OA were partially inserted into the lipid membrane. By contrast, annular structures similar to those previously reported by several groups [33–35] were present in the L_α_ domains (Figure 3B). In the absence of GM1, the accumulation of OA on the L_β_ domains was not observed (Figure 3E), while the annular rings on the L_α_ domains were still visible (Figure 3F). This was not surprising considering that GM1 resides in the L_β_ domains and the L_α_ phase is not modified by the absence of GM1. Small oligomeric assemblies were not present on the SLBs surface after the administration of OB, as clearly shown in the images acquired at higher lateral resolution (Figure 3C, 3D, 3G, 3H). In this case, both the L_β_ and L_α_ domains appeared essentially intact and did not display the presence of oligomers in the L_β_ phase (with the exception of rare large aggregates in GM1-enriched SLBs), or of annular structures in the L_α_ phase. The total distributions of height and width values of the globular and annular structures found in SLBs after exposure to HypF-N oligomers are shown in Figure 4. The corresponding mean values are summarized in Table I.

**Table 1:** Summary of the height and width values (means ± SD) of both globular oligomers and annular rings in both GM1-enriched and GM1-depleted SLBs

	+GM1		−GM1	
	Globular oligomers	Annular rings	Globular oligomers	Annular rings
height (nm)	2.0±0.5	0.4±0.2	-	0.4±0.2
width (nm)	34±8	19±4	-	19±4

The width values were measured as the widths at half height for globular oligomers and as peak to peak distances for annular rings.

### The negatively charged sialic acid residue of GM1 contributes to the interaction between HypF-N oligomers and lipid bilayers

**Figure 5:**
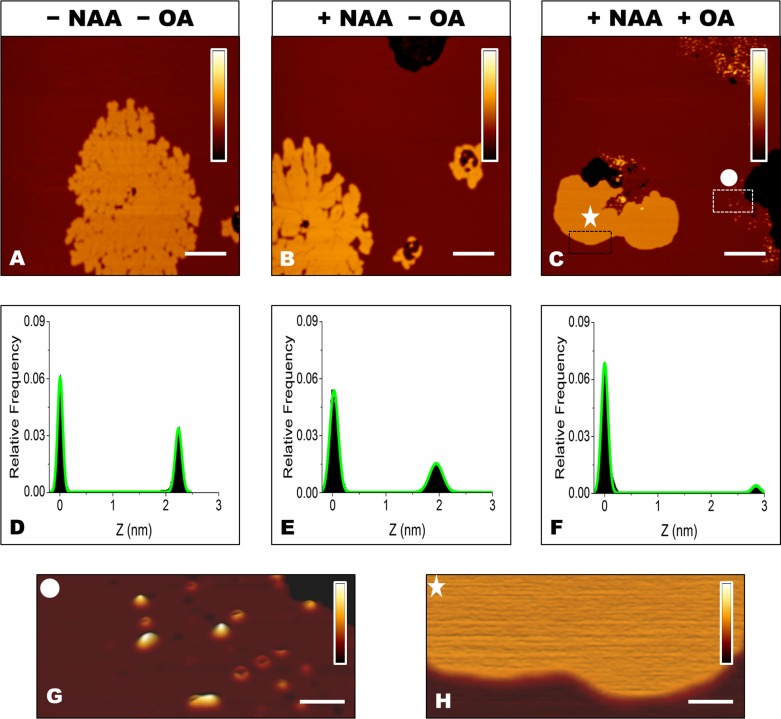
**A.**, **B.**, **C.** SLBs containing 5.0% GM1 before **A.**and after **B.**, **C.** treatment with NAA, and before **A.**, **B.** and after **C.** administration of 12 μM OA. The colour bars correspond to a *Z* range of 8.0 nm. Scale bars: 1.0 μm. **D.**,**E.**,**F.** Height distributions taken from the images shown in panels A, B and C, respectively, fitted to the sum of two Gaussian functions (green lines). **G.**, **H.** Magnification of the rectangular areas marked in panel C. After removal of the negatively charged sialic acid, protein aggregates are present in the L_α_ domain **G.**, but not in L_β_ domains **H.**. The colour bars correspond to a Z range of 5.0 nm. Scale bars: 100 nm.

We have previously reported that the sialic acid moiety of GM1 is essential for GM1-mediated toxicity of OA [16]. To better assess its role in the interaction between OA/OB oligomers and our supported lipid bilayers, GM1-enriched SLBs were treated with neuraminidase (NAA), which catalyses the removal of the sialic acid moiety from gangliosides (see Materials and methods for details). GM1-enriched SLBs presenting the two typically distinct lipid phases were observed in the absence of NAA treatment (Figure 5A). After treatment with NAA for 30 min, the membrane structure was maintained, except for the presence of a few holes, due to the local removal of SLB patches following the multiple rinsing procedure (Figure 5B). Indeed, similar damages of SLBs were also induced by consecutive rinsing steps in the absence of NAA. The height distribution after NAA treatment showed a slight decrease in ΔZ, from 2.2±0.2 nm, before NAA treatment, to 1.9±0.3 nm, after NAA treatment (Figure 5D, 5E).

ΔZ increased to 2.9±0.5 nm in NAA-treated SLBs exposed to OA (Figure 5C, 5F). In this case, aggregates mostly characterized by the annular shape were found on the L_α_ phase (Figure 5G), whereas no aggregates were observed on the L_β_ phase (Figure 5H). This scenario resembles that imaged in the absence of GM1 (Figure 3E, 3F), suggesting that the sialic acid moiety is responsible for the differences observed between GM1-enriched and GM1-free membranes.

### Interaction between toxic and non-toxic HypF-N oligomers and neuronal cells

**Figure 6:**
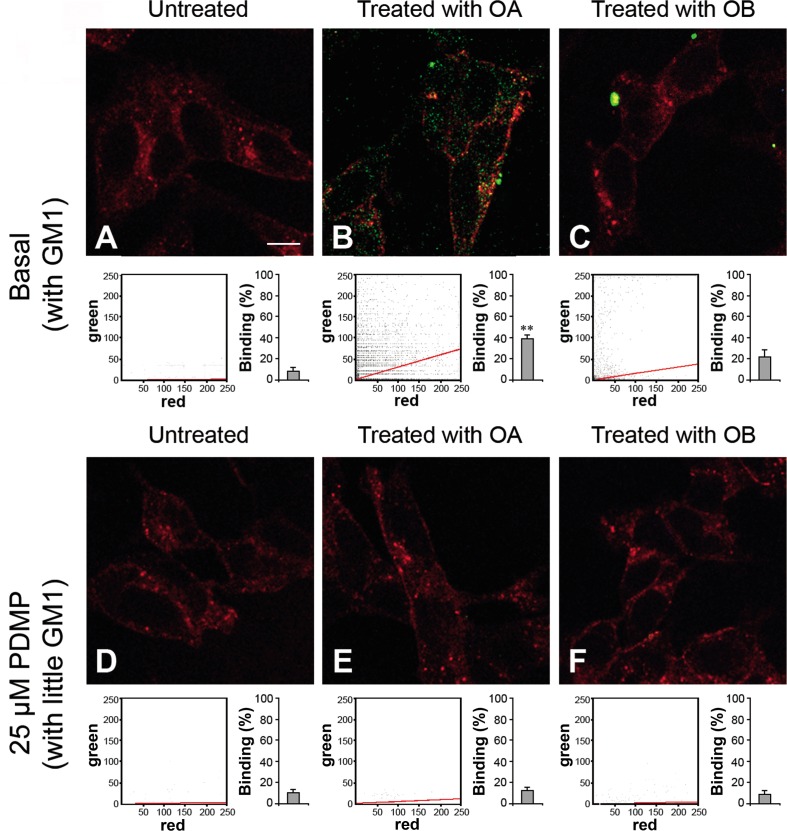
Representative confocal scanning microscopy images of the apical sections of neuroblastoma SH-SY5Y cells with basal **A., B., C. or reduced D., E., F. GM1 content.** The images were acquired on untreated **A.**, **D.**, OA-treated **B.**, **E.** or OB-treated **C.**, **F.** cells, respectively. The cells were exposed for 1.0 h to OA or OB (12 μM, monomer equivalent concentration). Red and green fluorescence indicates the cell membranes and the oligomers, respectively. The cytofluorograms of the three apical sections associated with the various images show the green *versus* red fluorescence intensity (as pixel intensity). The histograms show the percentage of co-localisation on regions of interest (12-13 cells) using the ImageJ (NIH, Bethesda, MD, USA) and JACOP plugin (rsb.info.nih.gov) software. Data are expressed as mean ± S.D. (*n* = 4 per group). Double asterisk: p ≤ 0.01 with respect to cells with basal GM1 content. Scale bars: 15.0 μm.

To assess whether our observations could be extended to a cellular system, we studied the interaction between HypF-N oligomers and human neuroblastoma SH-SY5Y cells. In particular, we performed a confocal microscopy analysis both on normal cells containing a physiological level of GM1, comparable to that of our SLBs containing GM1, and on cells treated with 25 μM D-threo-1-phenyl-2-decanoylamino-3-morpholino-1-propanol (PDMP), a glucosylceramide synthase inhibitor that reduces the GM1 content of the plasma membrane by blocking its biosynthesis [36]. Both basal and PDMP-treated cells were exposed to 12 μM (monomer equivalent) OA or OB and the degree of co-localization of the oligomers (green channel) with the cell membrane (red channel) was assessed by measuring the Pearson correlation coefficient (PCC) (Figure 6). The images were scanned at apical planes to detect oligomers interacting with the cell surface rather than internalized inside the cells. A large number of OA bound to the plasma membrane was found in OA-exposed cells with basal GM1 content (Figure 6B), whereas the same oligomers were undetectable in PDMP-treated cells incubated with OA (Figure 6E). By contrast, cells containing basal GM1 levels exposed to OB recruited a small number of large aggregates (Figure 6C) and the PDMP-treated cells did not bind oligomers (Figure 6F).

## DISCUSSION

A great deal of experimental evidence indicates that the interplay between oligomers arising from misfolded proteins/peptides and lipid membranes has a primary role in defining the cytotoxicity cascade associated with protein aggregation [5, 26, 37, 38]. It has also been reported that anionic lipids in the cell membranes promote the aggregation process of amyloidogenic peptides [39–42]. At the same time, other studies indicate that the toxicity of protein misfolded oligomers is directly related to their ability to affect the physiological functions of the cell membrane [33, 43–46]. In particular, in the last few years, great interest has been focused on the role of the negatively charged GM1 molecule, both as a nucleator of peptide/protein aggregation, as a key interaction site of pre-formed oligomers, and as a determinant of protein oligomer toxicity [16, 27–29, 47–51]. An interesting exemplification of the intimate interplay between oligomer structure and cell membrane composition is offered by the behaviour of the amyloid-forming protein domain HypF-N. It has been shown that the toxicity of HypF-N oligomers depends on both the oligomer structure and the physicochemical properties of the lipid membrane arising from its lipid composition [14, 16, 17, 19]. Only the oligomeric form here called OA appeared to be toxic to cultured neuronal cells, cultured primary neurons and whole animal models, resembling the effects of toxic Aβ oligomers, while the oligomeric form here called OB was not found to significantly affect cell viability and animal cognitive abilities [14–16, 18]. The toxicity of OA could be reduced or enhanced by depleting or increasing the content of membrane GM1 in neuroblastoma cells, respectively, whereas the non-toxic OB became toxic to GM1-enriched cells [16]. These data strongly suggested that GM1 is a main mediator of oligomer toxicity and agreed with similar data obtained with two types (toxic and non-toxic) of Aβ oligomers leading to propose a generalisation of these conclusions [16].

Since the interaction between HypF-N oligomers and the cell membrane is a central event of oligomer-induced cytotoxicity, we initially used SLBs as a model system to mimic the interaction between cell membranes and HypF-N oligomers. All the variables playing some role in this interaction were considered by studying the behaviour of the two isoforms, OA and OB, and using GM1-free SLBs, or SLBs containing a GM1 concentration similar to that found in the plasma membrane of neuronal cells. This approach allowed the contribution of the lipid component of the plasma membrane to be evaluated independently of that of the proteins populating the membrane itself and the oligomer-membrane interaction to be monitored with unprecedented resolution. In fact, we imaged the morphology of the oligomers and their mode of interaction with the membrane; at the same time it was possible to discriminate between the ordered and disordered lipid phases of the bilayer, recalling some features found in the lipid rafts and in the surrounding membrane bilayer in cells, respectively. The results obtained by the AFM analysis were compared with those derived by confocal microscopy of cell membranes to parallel the data obtained with the first approach with those obtained with cultured cells.

The main findings of the present study can be summarized in the following four points. The first important observation is that OA and OB interact very differently with SLBs; in particular, OA display greater affinity to the lipid membrane, confirming the data obtained here and previously with neuroblastoma cells [16]. The high resolution images show that OA bound more extensively and deeply than OB to the lipid bilayer (Figure 3A-3D). Moreover, the interaction between OA (but not OB) and SLBs was found to induce structural changes of the overall bilayer structure, with a significant increase in ∆Z (Figure 2). This effect cannot be due to an increase in thickness of the L_β_ phase domains, in which the lipid chains are already stretched. Furthermore, higher magnification images excluded that one or more layers of oligomers were formed on the top of these regions. The formation of multilayer domains was also excluded by a topographical analysis; in fact, the average value of ∆Z was increased by 1.5 nm in the presence of GM1 and by 1.1 nm in its absence, which is an insufficient increase to justify the formation of a second lipid bilayer. All these considerations lead us to conclude that the observed variation of ∆Z results from some decrease in thickness of the L_α_ phase domains, likely due to the promotion of a thinner, possibly interdigitated, phase, in agreement with previous findings obtained on single phase SLBs [52, 53].

A second important observation is that OA interact with both the L_α_ and L_β_ phase domains of the membrane with two different morphologies (Figure 3A, 3B). However, GM1 mediates the interaction only with the L_β_ phase domain as GM1 resides only in this phase and its absence in GM1-free SLBs causes the loss of the OA-L_β_ interaction while maintaining the OA-L_α_ association (Figure 3E and 3F). Experiments performed on GM1-enriched SLBs treated with NAA showed that this interaction is also suppressed by removing the sialic acid residue of GM1, clearly indicating that the high affinity between OA and GM1-enriched L_β_ phase domains is driven mainly by the sialic acid moiety, possibly through electrostatic interactions (Figure 5).

A third finding is that only the oligomers interacting with the L_β_ phase domain of the SLBs appear to be responsible for cytotoxicity since their amount correlates with that of the oligomers bound to the membrane of neuroblastoma cells and the ensuing cytotoxicity [16]. Indeed, the level of interaction found here followed the ranking OA-L_β_ > OB-L_β_ > OA/OB-L_β_ in GM1-free SLBs, which parallels that found in neuroblastoma cells: OA-cell > OB-cell > OA/OB-cell in GM1-depleted cells. Such a ranking matched that of the observed toxicity: OA cytotoxicity > OB cytotoxicity > OA/OB cytotoxicity to GM1-depleted cells [16]. Interestingly, OA are no longer toxic to GM1-depleted cells and this parallels our finding in GM1-free SLBs, where only the OA-L_β_ interaction was lost, whereas the interaction with the L_α_ phase domain was maintained. In other words, a parallel does exist between (i) HypF-N oligomers bound to the L_β_ phase domain of SLBs, (ii) HypF-N oligomers bound to the cell membrane and (iii) oligomer cytotoxicity.

A fourth important finding emerging from our study is that no correlation appears to exist between OA that adopt a pore-like morphology upon binding to the L_α_ phase domain of the SLBs and any cytotoxicity. Indeed, OA bound to the L_α_ phase domain both in GM1-enriched and in GM1-depleted SLBs (Figure 3B, 3F), whereas their cytotoxicity was dramatically reduced only in GM1-depleted cells [16]. Moreover, a large distribution of OA was clearly detected in oligomer-exposed SH-SY5Y cells with basal GM1 content and the high level of co-localization indicated that the aggregates were present at the cell membrane level (Figure 6B). By contrast, OA displayed little affinity for PDMP-treated (GM1-depleted) cells (Figure 6E). Hence, the GM1-independent affinity of OA for the L_α_ phase domain of SLBs resulting in the insertion of annular structures in the lipid bilayer, is strongly reduced, if not completely missing, at the cell membrane level. This finding rules out that the cytotoxicity of OA is entrusted with the ability of these species to adopt a pore-like morphology in the cell membrane; rather it indicates that the spherical or disc-shaped oligomers bound to the L_β_ phase domain are the true toxic species.

The tendency of protein oligomers to interact with synthetic lipid membranes supports the idea that the interaction is not driven by the action of specific membrane protein targets but, possibly, is modulated by electrostatic interactions between the negatively charged GM1 molecules and the positive charge distribution within HypF-N oligomers and between the hydrophobic environment of the bilayer interior and by the hydrophobic groups on the surface of the protein oligomers. However, the possibility that intracellular calcium overload can in part be due to the interaction of oligomers with endogenous calcium-permeable channels or membrane receptors cannot be ruled out. In this context, several cell surface proteins have been considered as possible candidate receptors of Aβ oligomers, including APP [54], tumor-necrosis factor receptor-1 (TNFR1) [55], the receptor for advanced glycation end products (RAGE) [56], the non-infectious form of the prion protein (PrPc) [57], voltage-gated calcium channels [58] or ligand-gated calcium channels such as the glutamate N-methyl D-aspartate (NMDA) receptors and the α-amino-3-hydroxy-5-methyl- 4-isoxazolepropionic acid (AMPA) receptor [59, 60].

In conclusion, our findings, together with the toxicity data obtained with cultured cells [16], indicate that the importance of GM1 in mediating the cytotoxicity of HypF-N oligomers can be traced back to its ability to recruit protein aggregates to lipid raft domains of the cell membrane, a behaviour that appears mainly due to the negative charge of GM1 and does not require the participation of specific or generic protein components or the adoption of a well defined annular morphology by the oligomers. These data add to, and complement, those previously reported showing the key importance of GM1 and its clusters in the neuronal membrane not only as promoters of Aβ aggregation [27, 28], but also as binders of Aβ oligomers [29, 61], In the past years, the observation of annular amyloid structures on model membranes, as well as of an increased membrane permeability to Ca^2+^ ions in aggregate-exposed synthetic bilayers and cultured cells, led to the formulation of the “channel hypothesis”. This states that the altered intracellular calcium concentration in cells treated with protein misfolded oligomers is related to uncontrolled calcium fluxes resulting from the organization of non-specific pores in aggregate-exposed bilayers [34, 62–64]. In contrast with this view, and in agreement with another part of literature [43], our study indicates that the cytotoxicity of protein misfolded oligomers is related to a more general mechanism involving the destabilization and disassembly of the lipid membrane within the lipid raft regions, causing an influx of Ca^2+^ ions from the extracellular to the intracellular environment.

## MATERIALS AND METHODS

### Synthetic lipid vesicle preparation and SLBs formation

1,2-dioleoyl-sn-glycero-3-phosphocholine (DOPC,18:1), sphingomyelin (SM) (brain, porcine) and ganglioside GM1 (brain, ovine - sodium salt) were purchased from Avanti Polar Lipids (Alabaster, AL, USA). Cholesterol (Chol), chloroform and methanol were from Sigma-Aldrich (St Louis, MO, USA). Two different lipid mixtures were used for vesicle preparation, the first composed of DOPC/SM 2:1 (mol/mol) + 1.0% (mol) cholesterol and the second composed of DOPC/SM 2:1 (mol/mol) + 1.0% (mol) cholesterol + 5.0% (mol) GM1 (DOPC:SM:chol and DOPC:SM:chol:GM1, respectively). The phospholipid powder was dissolved in chloroform/methanol (2:1), according to the desired composition, and gently evaporated to dryness under nitrogen flux. Aliquots of the mixture were stored overnight under vacuum and resuspended in Milli-Q water at a lipid concentration of 0.5 mg/mL to form multilamellar vesicles (MLVs). MLVs suspensions were let to swell for 1.0 h at 60°C and then extruded 11 times through a polycarbonate membrane with 100 nm pores using a commercial extruder (Avanti Polar Lipids) at the same temperature, to form large unilamellar vesicles (LUVs). After cooling at room temperature, LUVs suspension was diluted 5 fold with Milli-Q water. Then, 40 μL of each suspension and 10 μL of a 10 mM CaCl_2_ solution were deposited onto a 1.0 cm × 1.0 cm freshly cleaved mica substrate. In order to get uniform bilayer coverage, the samples were stored 10 min at room temperature and then incubated 15 min at 60°C in a close chamber with 100% relative humidity. The samples were cooled at room temperature and, after 2.0 h, gently rinsed three times with Milli-Q water to remove excess vesicles from the liquid subphase before AFM measurements.

### Sialic acid removal

The removal of the sialic acid residues from DOPC:SM:chol:GM1 bilayers was achieved by treating the lipid bilayer with a neuraminidase (NAA) cocktail (117 mU/mL of *Vibrio cholerae* NAA and 33 mU/mL of *Arthrobacter ureafaciens* NAA, both purchased from Sigma-Aldrich) for 30 min at room temperature. The sample was rinsed three times with Milli-Q water before AFM imaging.

### Preparation of HypF-N oligomers

HypF-N was purified as previously described [14]. HypF-N oligomers were prepared, starting from frozen aliquots of the purified native protein, using two different aggregation conditions, as previously described [14]. In brief, for toxic OA preparation, stock aliquots of native HypF-N were diluted to 0.5 mg/ml in 50 mM acetate buffer containing 12% (v/v) trifluoroethanol (TFE), 2.0 mM dithiothreitol, at pH 5.5. After 4.0 h incubation at 25°C, the solution was centrifuged at 16,100 r.c.f. for 10 min and resuspended in PBS at 0.5 mg/mL concentration. Non-toxic OB were prepared by the same procedure, but using 20 mM trifluoroacetic acid (TFA), 330 mM NaCl, pH 1.7 as aggregation buffer.

### AFM imaging

AFM images were acquired by using a Nanowizard III (JPK Instruments, Germany) mounted on an Axio Observer D1 (Carl Zeiss, Germany) inverted optical microscope. V-shaped DNP silicon nitride cantilevers (Bruker, MA, USA), with a nominal spring constant 0.24 N/m, resonance frequency in air ranging from 40 kHz to 75 kHz and tip typical curvature radius of 20-60 nm were used. Intermittent contact mode AFM images were acquired in the constant-amplitude mode, working in water with an oscillating frequency of 10-20 kHz. The amplitude setpoint was kept above 70% of free oscillation amplitude in all cases. OA and OB were administered to the sample under the AFM head at a final concentration of 12 μM and left standing for 30 min. The difference in thickness between ordered (L_β_) and disordered (Lalpha) lipid domains (ΔZ) was determined by considering image height distributions: the values of height per each one of the 512×512 image data points were calculated by using the JPK Data Processing software (JPK Instruments, Germany). Raw data were plotted by using Origin (OriginLab, MA, USA) and the distributions were fitted to the sum of two Gaussian functions. The ΔZ value associated with each image was the separation between the peaks of the two Gaussian curves. This procedure was repeated for at least 10 different images for each experiment.

### Oligomer binding to the cell membrane

Human neuroblastoma SH-SY5Y cells (A.T.C.C. Manassas, VA, USA) were cultured in Dulbecco's Modified Eagle's Medium (DMEM), F-12 Ham with 25 mM HEPES and NaHCO_3_ (1:1) supplemented with 10% fetal bovine serum (FBS), 1.0 mM glutamine and 1.0% penicillin and streptomycin solution in a 5.0% CO_2_ humidified atmosphere at 37°C. The cells were seeded on glass coverslips and cultured with the cell culture medium described above (basal GM1 content) or supplemented with 25 μM D-threo-1-phenyl-2-decanoylamino-3-morpholino-1-propanol (PDMP) for 48 h at 37°C (low GM1 content). Then the cells were treated for 1.0 h with 12 μM (monomer equivalent) OA or OB and, after incubation, counterstained with 5.0 μg/mL Alexa Fluor 633-conjugated wheat germ agglutinin. The presence of oligomers was detected with 1:1000 diluted rabbit polyclonal anti-HypF-N antibodies and subsequently with 1:1000 diluted Alexa Fluor 488-conjugated anti-rabbit secondary antibodies. Fluorescence emission was detected after double excitation at 488 nm and 633 nm by a TCS SP5 scanning confocal microscopy system (Leica Microsystems, Mannheim, Germany) equipped with an argon laser source. A series of 1.0 μm thick optical sections (1024 × 1024 pixels) was taken through the cell depth for each sample using a Leica Plan Apo 63× oil immersion objective and three apical sections were projected as a single composite image by superimposition. Oligomer and apical cell membrane co-localisation was estimated for regions of interest in 12-13 cells, in four different experiments, using ImageJ and JACOP plugin (http://www.rsb.info.nih.gov) software and expressed as fraction of oligomer binding by the Pearson's correlation coefficient (PCC).
